# Adjuvant effects of combination monophosphoryl lipid A and poly I:C on antigen-specific immune responses and protective efficacy of influenza vaccines

**DOI:** 10.1038/s41598-023-39210-6

**Published:** 2023-07-28

**Authors:** Chau Thuy Tien Le, So Yeon Ahn, Thi Len Ho, Jueun Lee, Dong-Ha Lee, Hye Suk Hwang, Sang-Moo Kang, Eun-Ju Ko

**Affiliations:** 1 Interdisciplinary Graduate Program in Advanced Convergence Technology and Science, Jeju National University, Jeju, 63243 Republic of Korea; 2 Center for Inflammation, Immunity and Infection, Institute for Biomedical Sciences, Georgia State University, Atlanta, GA 30303 USA; 3 College of Veterinary Medicine and Veterinary Medical Research Institute, Jeju National University, Jeju, 63243 Republic of Korea; 4 Department of Biomedical Science, College of Life Science and Industry, Sunchon National University, Suncheon, 57922 Republic of Korea

**Keywords:** Adaptive immunity, Infectious diseases, Vaccines

## Abstract

Toll-like receptor (TLR) agonists improve vaccine immunogenicity and efficacy, but they are currently unlicensed as adjuvants in influenza vaccines. This study aimed to investigate whether a combination of monophosphoryl lipid A (MPL, a TLR4 agonist) and polyriboinosinic polyribocytidylic acid (poly I:C, a TLR3 agonist) can enhance the protective efficacy of an inactivated A/Puerto Rico/8/1934 (A/PR8) H1N1 influenza vaccine against homologous influenza infection and minimize illness outcomes. Results showed that combination MPL and poly I:C adjuvanted influenza vaccination increased the production of antigen-specific antibodies, decreased the levels of cytokines and cellular infiltrates at the infection sites, and induced significant memory T and B cell responses in mice. The results of this study suggest that the combination of MPL and poly I:C can be developed into a possible adjuvant for enhancing the efficacy of influenza vaccines.

## Introduction

Influenza infections are responsible for an average of 250,000–500,000 respiratory deaths annually, predominantly caused by the A(H1N1) 2009 pandemic (pdm09) subtype (in people < 65 years) and A(H3N2) subtype viruses (in people ≥ 65 years)^1^. Vaccination is the most effective strategy for preventing influenza virus infections. Widely used seasonal influenza vaccines are inactivated whole-virus, split-virus, and protein subunit vaccines, but the efficacy of influenza vaccines is suboptimum^2–4^. Moreover, these vaccines are ineffective in conferring cross-protection against antigenically different influenza viruses; thus, annual influenza vaccination is required, leading to an economic burden on the healthcare system and society^4,5^.

Innate immune responses to pathogens are commonly induced by the recognition of specific patterns of pathogens via toll-like receptors (TLRs), which are expressed on most innate immune cells. Interactions between TLRs and their ligands activate downstream factors of TLR-dependent signaling pathways, such as nuclear factor-kappa B, activator protein-1, and interferon (IFN) regulatory factor-3/7. The release of pro-inflammatory cytokines and infiltration of innate immune cells into the infection site further initiate antigen-specific adaptive immunity^6^. Therefore, TLR agonists are promising candidates as vaccine adjuvants to improve vaccine efficacy and enhance mucosal and humoral immunity.

Monophosphoryl lipid A (MPL) is a TLR4 agonist that stimulates immune responses via myeloid differentiation factor 88 (MyD88) and Toll-IL-1R domain-containing adaptor-inducing IFN-β factor (TRIF)-dependent pathways; at high doses (10–50 μg), MPL is used as an adjuvant for respiratory syncytial virus vaccines^7^. Synthetic double-stranded RNA polyriboinosinic polyribocytidylic acid (poly I:C) is a TLR3 agonist that induces the TRIF-dependent signaling pathway, leading to the release of pro-inflammatory cytokines and local immune responses. Poly I:C-adjuvanted influenza vaccines elicit a high anti-hemagglutinin (HA) response in nasal wash and enhance the production of IgG isotype antibodies, resulting in increased protection against influenza viral infections^8^. In previous studies, both MPL and poly I:C have been used with a relatively high dose; 10 μg to 50 μg of MPL and 50 μg of Poly I:C for mice, and up to 1 mg per each adjuvant for Rhesus Macaques immunization^9–12^. A combination adjuvant composed of low doses of MPL (1 μg) and poly I:C (10 μg) positively promotes innate and specific adaptive immune responses to ovalbumin^13,14^. In this study, we aimed to investigate whether the combination of MPL and poly I:C enhances the protective efficacy of an inactivated A/Puerto Rico/8/1934 (A/PR8) H1N1 influenza vaccine in comparison with nonadjuvanted or individual adjuvanted vaccines after homologous viral challenge and elicit heterosubtypic antibody production against the A/Hong Kong/1/68 (A/HK) H3N2 virus.

## Results

### MPL + poly I:C-adjuvanted iPR8 vaccine enhances antigen-specific antibody production

**Figure 1:**
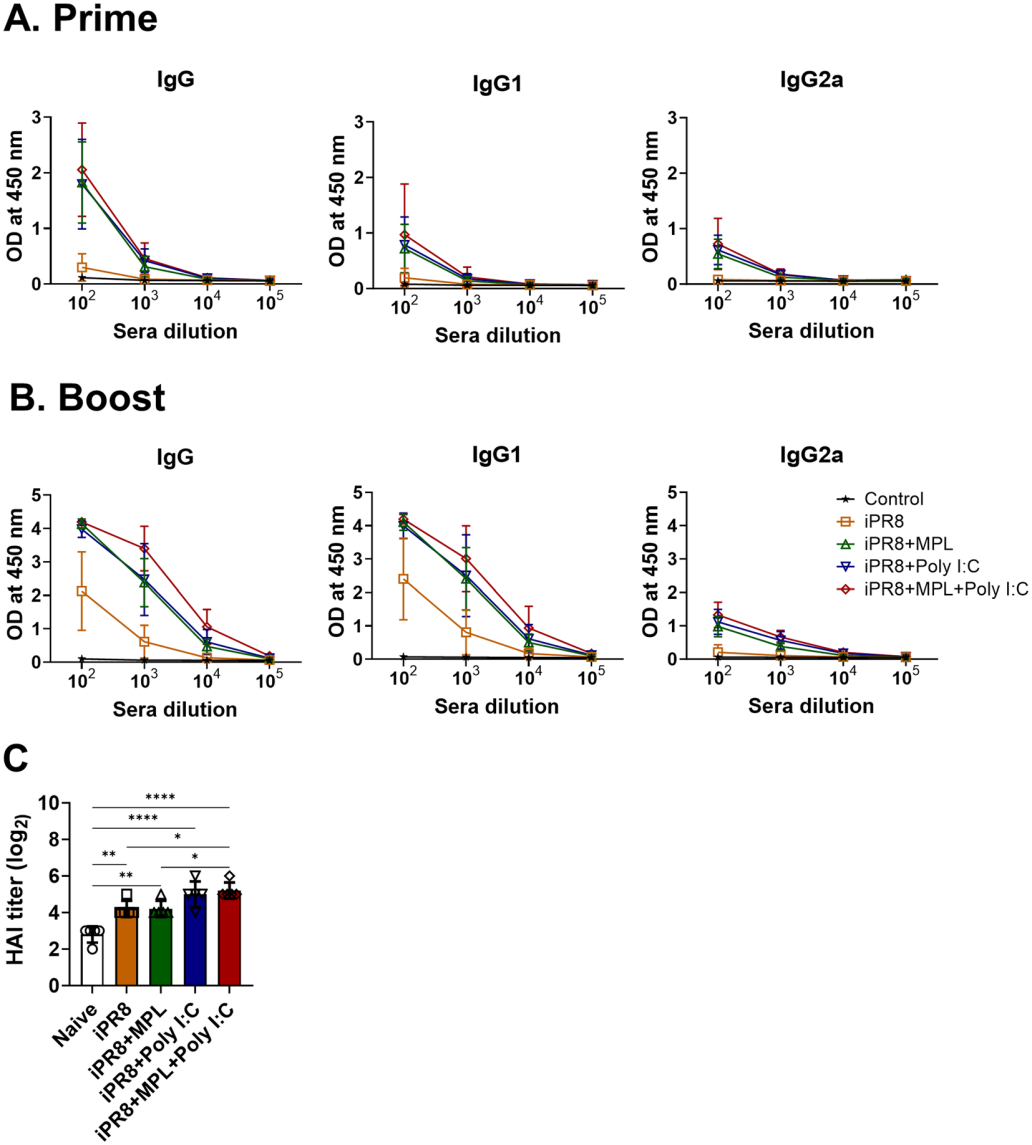
Antibody production after inactivated A/PR8 vaccine with or without adjuvants. BALB/c mice (n = 10) were intramuscularly immunized two times with a 3 weeks interval, with inactivated A/PR8 (1 μg) with or without adjuvants (1 μg of MPL, 10 μg of poly I:C or MPL 1 μg + poly I:C 10 μg). (**A** and **B)** A/PR8 (H1N1)-specific antibody levels in sera collected at 2 weeks after prime and boost immunizations, respectively. (**C**) Hemagglutination inhibition (HAI) titers of boost sera against homologous A/PR8 (H1N1). All results are presented as mean ± standard deviation (SD) with individual dots. Statistical analysis was performed using two-way ANOVA and Tukey’s post-multiple comparison tests. *P < 0.0332, **P < 0.0021, ****P < 0.0001.

To determine the effects of the adjuvanted influenza vaccines, we intramuscularly injected BALB/c mice (n = 10/group) with an inactivated A/PR8 (H1N1) vaccine (iPR8, 1 μg) alone or in combination with an adjuvant (either MPL 1 μg, poly I:C 10 μg, or MPL 1 μg + poly I:C 10 μg) two times with a 3 weeks interval. Sera were collected at 2 weeks after each immunization, and A/PR8-specific isotype antibodies were measured using ELISA. The A/PR8-specific HAI titers at 2 weeks post boost immunization were determined by performing an HAI assay. Compared with the nonadjuvanted vaccines, the adjuvanted iPR8 vaccines induced higher levels of virus-specific total IgG, IgG1, and IgG2a isotype antibodies after the prime immunization (Fig. 1A). The levels of serum anti-A/PR8 antibodies were significantly increased by boost dose of iPR8 with MPL + poly I:C compared with iPR8 with MPL or poly I:C (Fig. 1B). iPR8 alone vaccination elicited the production of Th2-induced IgG1 isotype antibodies but not Th1-induced IgG2a isotype antibodies after boost immunization. The mice in the MPL- or poly I:C-adjuvanted vaccine group presented a significant increase in the levels of antigen-specific antibodies compared to the unadjuvanted vaccine group. In addition, the HAI titers in immune sera were higher in the mice from the poly I:C- or MPL + poly I:C-adjuvanted group than in the mice from the MPL-adjuvanted or nonadjuvanted group (Fig. 1C).

### MPL + poly I:C-adjuvanted iPR8 vaccine enhances protection against the lethal homologous influenza infection

**Figure 2:**
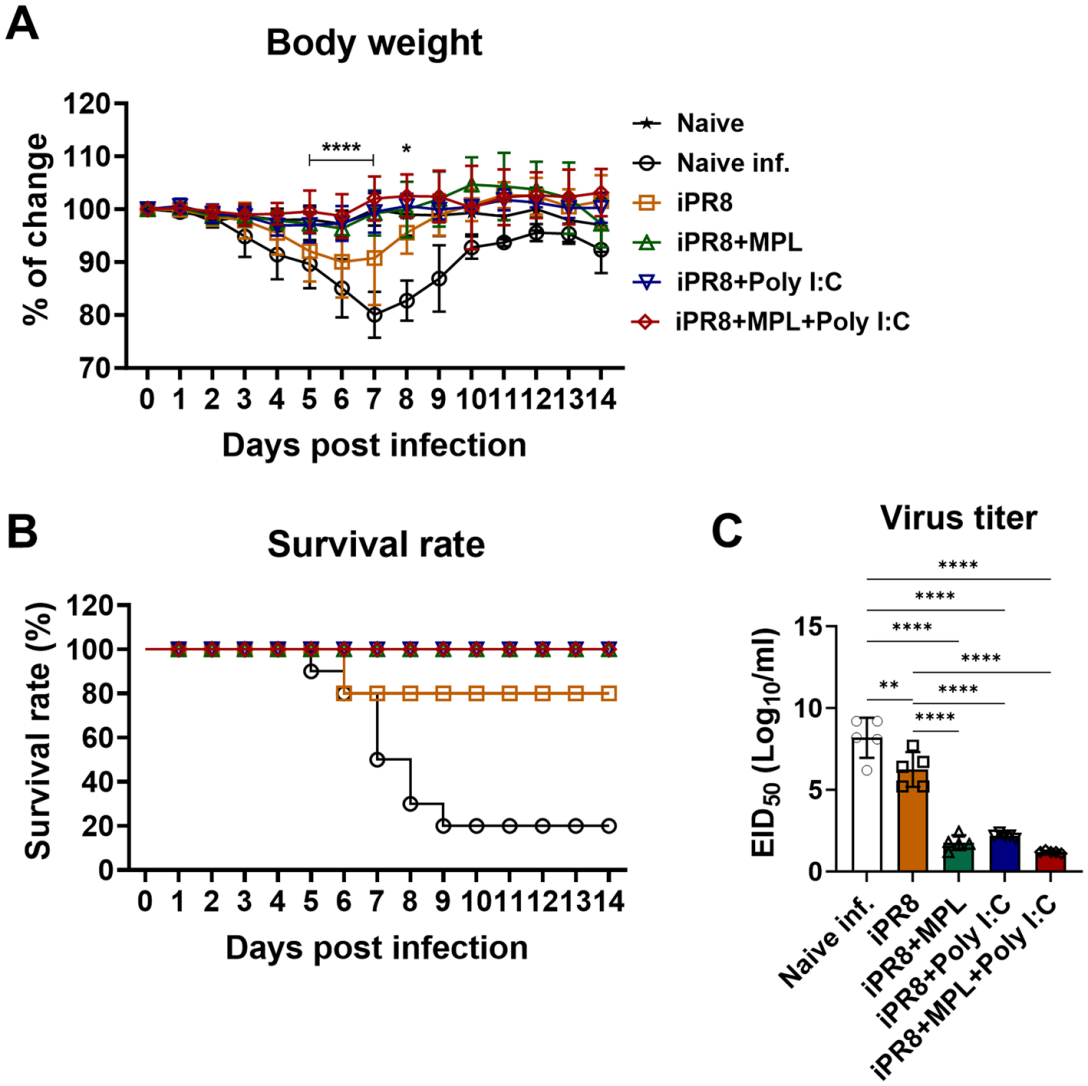
Protective efficacy of the adjuvanted vaccines in the immunized mice after the lethal influenza virus challenge. Immunized BALB/c mice (n = 10) were challenged with a lethal dose of homologous A/PR8 (1.5 × LD_50_) after 3 weeks of boost immunization. Body weight (BW) (**A**) and survival rates (**B**) of the challenged mice (n = 5) were measured for 14 days after viral infection, and percentages of changes were calculated based on BW at day 0. For statistical analysis, two-way ANOVA and Tukey’s post-multiple comparison tests were performed. *p < 0.0332, and ****p < 0.0001 between iPR8 and iPR8 + MPL + Poly I:C groups. (**C**) Lung homogenates (n = 5) were harvested at day 7 post infection, and lung viral titers were measured. Statistical analysis was performed using one-way ANOVA and Tukey’s post-multiple comparison tests. **P < 0.0021, ***P < 0.0002, ****P < 0.0001. All results were shown in mean ± standard deviation (SD) with individual dots.

At 3 weeks after boost vaccination, the naïve and vaccinated mice were challenged with a lethal dose of A/PR8 (1.5 × LD_50_) to investigate the protective efficacy of the vaccine with the adjuvants. To rule out the non-specific protective effects of adjuvant itself, mice were immunized with adjuvants only (MPL, Poly I:C, or MPL + Poly I:C without iPR8) and evaluated antibody production and protection against the virus challenge. The adjuvant only immunization could not provide anti-viral responses as well as any antigen-specific antibody production (Supplementary Fig. 1). The adjuvanted vaccines showed effective protection against a lethal dose of A/PR8 infection, as evidenced by the no loss in body weight (BW) and 100% survival rate of the challenged mice, which is similar to those of the uninfected naïve mice. However, the naïve mice displayed severe weight loss (19%–25%) at 7 days post challenge (dpi) and 20% survival rate (Fig. 2A,B). Even survived mice exhibited severe illness such as near 20% weight loss, hunched posture, and scruffy hair coat during the infection. The iPR8-vaccinated mice displayed a slight decrease in BW (10–20%) and 80% survival rate at 6 days post challenge. After 9 days of challenge, the weights of the iPR8-vaccinated mice recovered to normal, but those of the naïve infected mice did not (Fig. 2A,B).

To investigate whether immunization with inactivated influenza vaccine together with adjuvants could prevent viral replication, we collected lung samples of vaccinated mice at 7 days after homologous challenge to measure viral titers (Fig. 2C). The nonvaccinated and iPR8-only-vaccinated mice showed significantly high levels of lung viral titers at 7 dpi, whereas the mice in the single-adjuvanted and MPL + poly I:C-adjuvanted groups exhibited significantly lower titers, suggesting better viral control in the lungs. In particular, the mice in the MPL + poly I:C-adjuvanted group showed the detection limit of viral titers. These data suggest that the MPL- and poly I:C-adjuvanted iPR8 vaccines provided complete protection against homologous infection.

**Figure 3:**
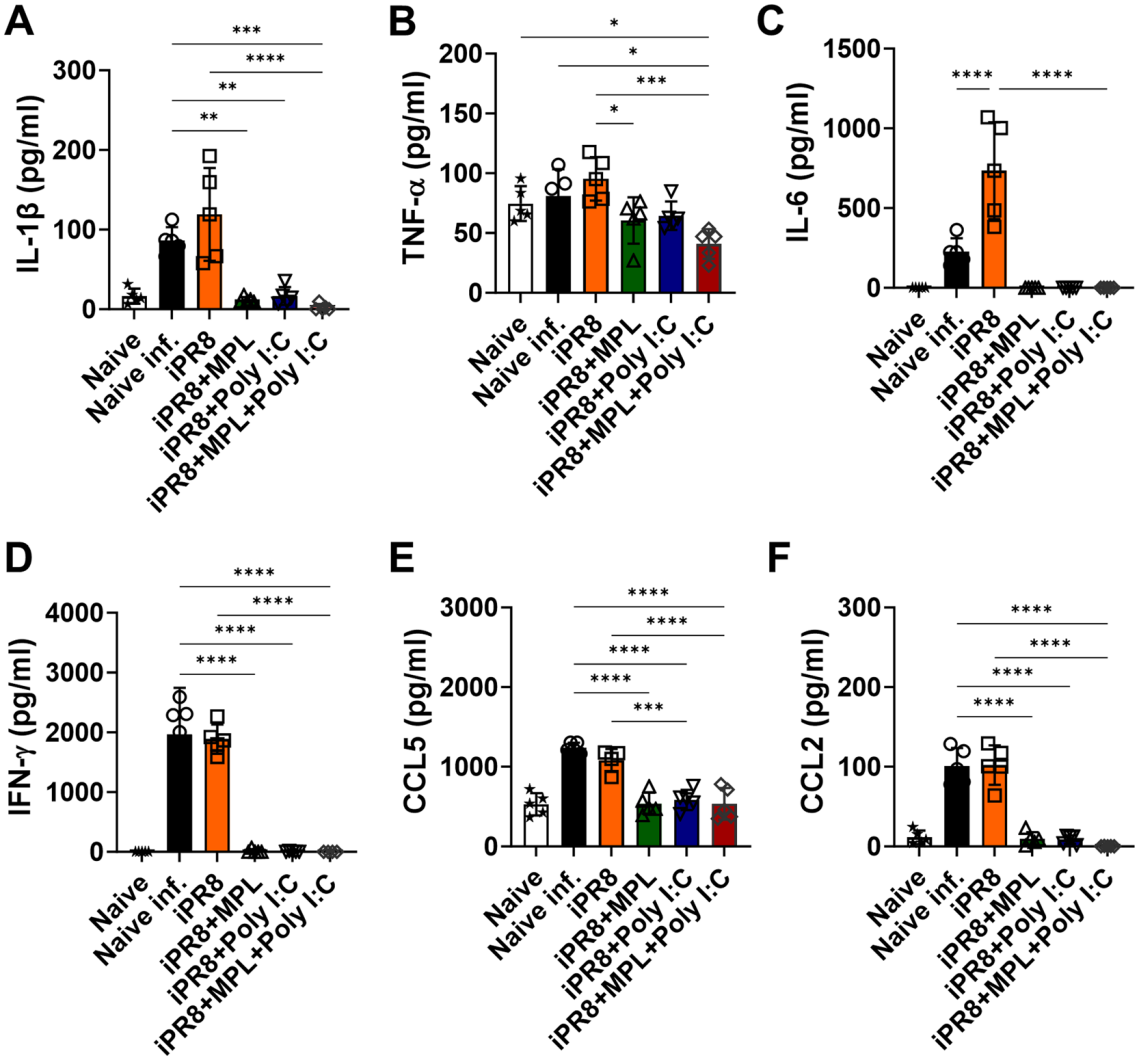
Cytokines and chemokines released into lung extracts in BALB/c mice after homologous A/PR8 challenge. Lung samples of the vaccinated BALB/c mice (n = 5) were harvested at 7 days post challenge. Cytokine and chemokine levels were measured using ELISA. All results are presented as mean ± standard deviation (SD) with individual dots. Statistical analysis was performed using one-way ANOVA and Tukey’s post-multiple comparison tests. *P < 0.0332, **P < 0.0021, ***P < 0.0002, ****P < 0.0001.

Cytokine storms with high levels of pro-inflammatory cytokines elicit inflammatory cell recruitment to the infection site; however, the cytokine levels in the lungs after infection are inversely correlated with viral loads in the lungs and positively correlated with disease severity during influenza infection^15,16^. We measured the levels of cytokines and chemokines in lung extracts harvested from the mice in the vaccinated groups at 7 dpi (Fig. 3). Among the mice in this study, the naïve infected and iPR8-only-vaccinated mice showed the highest production of cytokines (IL-1β, TNF-α, IL-6, and IFN-γ) and chemokines (CCL2 and CCL5) after lethal viral challenge. The adjuvanted immunizations, however, exhibited low or no cytokine/chemokine production, indicating that the adjuvants increased the protective efficacy of the vaccine.

To further confirm the protective efficacy of the adjuvant combination against the influenza virus infection, lung samples were harvested, processed, and stained with hematoxylin and eosin. Severe lung inflammation was observed in naïve and iPR8 immunized groups 7 days post virus challenge. Meanwhile MPL- and poly I:C-adjuanted groups exhibited moderate levels of lung inflammation, MPL + poly I:C-adjuvanted group did not display lung histopathology (Supplementary Fig. 2).

### MPL + poly I:C-adjuvanted iPR8 vaccine prevents inflammatory cell infiltrates after A/PR8 viral infection

**Figure 4:**
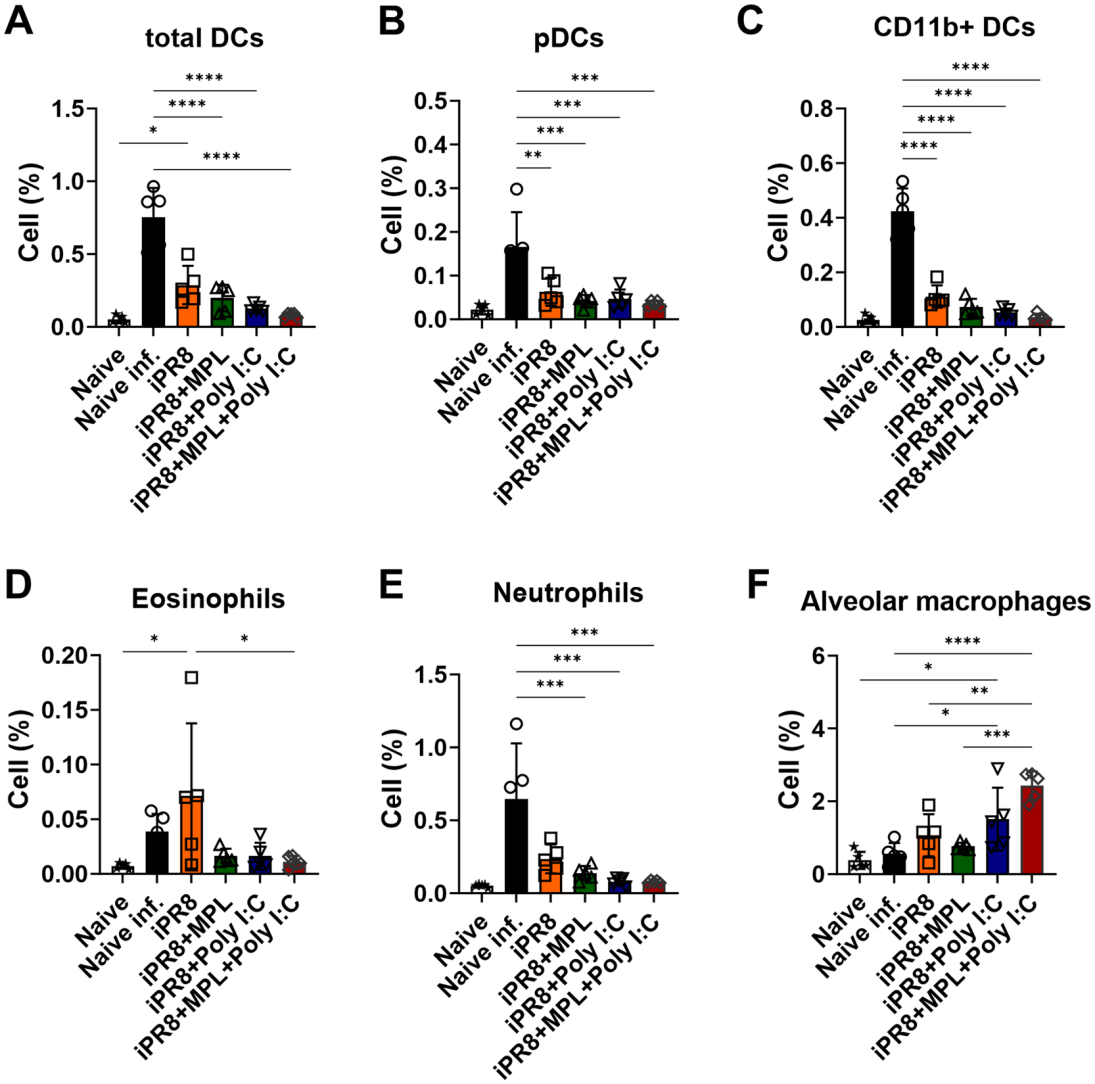
Inflammatory cell infiltration into the lungs after homologous viral infection. Immunized BALB/c mice (n = 5) were infected with A/PR8 (1.5 × LD_50_) after 3 weeks of boost immunization. Lung samples were harvested at day 7 post infection and then cell phenotypes were investigated by flow cytometry. All results were shown in mean ± standard deviation (SD) with individual dots. Statistical analysis was performed using one-way ANOVA and Tukey’s post-multiple comparison tests. **P < 0.0021, ***P < 0.0002, ****P < 0.0001.

Cellular infiltrates in the lungs of immunized BALB/c mice were observed 7 days after homologous viral challenge to assess a possible correlation with the protective efficacy of the vaccines (Fig. 4). Inflammatory cells play a role in protection against invading pathogens, but they also contribute to lung inflammation, leading to severe illness outcomes upon viral infection^17,18^. Consistent with the high production of pro-inflammatory cytokines (Fig. 3), inflammatory cells, such as eosinophils, neutrophils, and DCs were recruited to the lungs of the naïve mice after viral infection. More inflammatory cells were observed in the lungs of the mice from the single-adjuvanted groups than in those of the mice from the MPL + poly I:C-adjuvanted group, but the difference was not significant (Fig. 4A–E). Alveolar macrophages (AMs) are the first line of defense against pathogens invading the airway. The percentages of AMs were maintained in the mice from the iPR8-only, poly I:C-adjuvanted, and MPL + poly I:C-adjuvanted groups, whereas the mice from the naïve infected and MPL-adjuvanted groups showed decreased AM populations in the lungs (Fig. 4F). These data suggest that the MPL + poly I:C adjuvant in the inactivated vaccine effectively prevented the infiltration of inflammatory cells into the lungs upon influenza infection.

### Induction of memory T cell populations and antigen-specific T-cell responses as well as memory B cell responses by the intramuscular administration of vaccine adjuvanted with MPL + poly I:C

**Figure 5:**
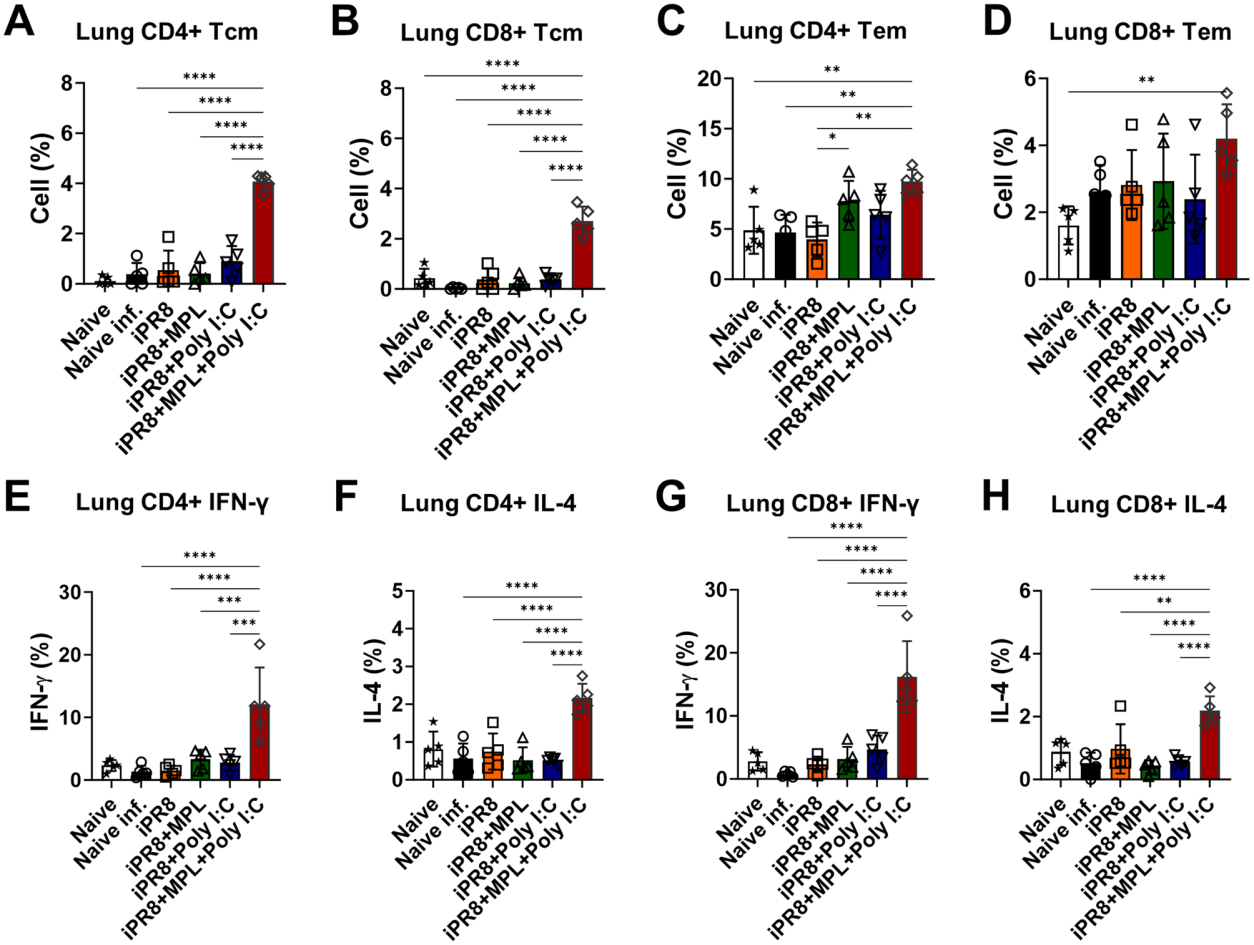
Memory T cell populations and iPR8-specific IFN-γ and IL-4-producing T cells in the lungs of immunized mice. Vaccinated mice were infected with a lethal dose of A/PR8 (1.5 × LD_50_) after 3 weeks of vaccination. Lung samples were harvested 7 days after virus challenge and then stimulated with inactivated PR8 antigen for 5 h. Cell phenotypes were evaluated using flow cytometry. All results are presented as mean ± standard deviation (SD) with individual dots. Statistical analysis was performed using one-way ANOVA and Tukey’s post-multiple comparison tests. **P < 0.0021, ***P < 0.0002, ****P < 0.0001.

**Figure 6:**
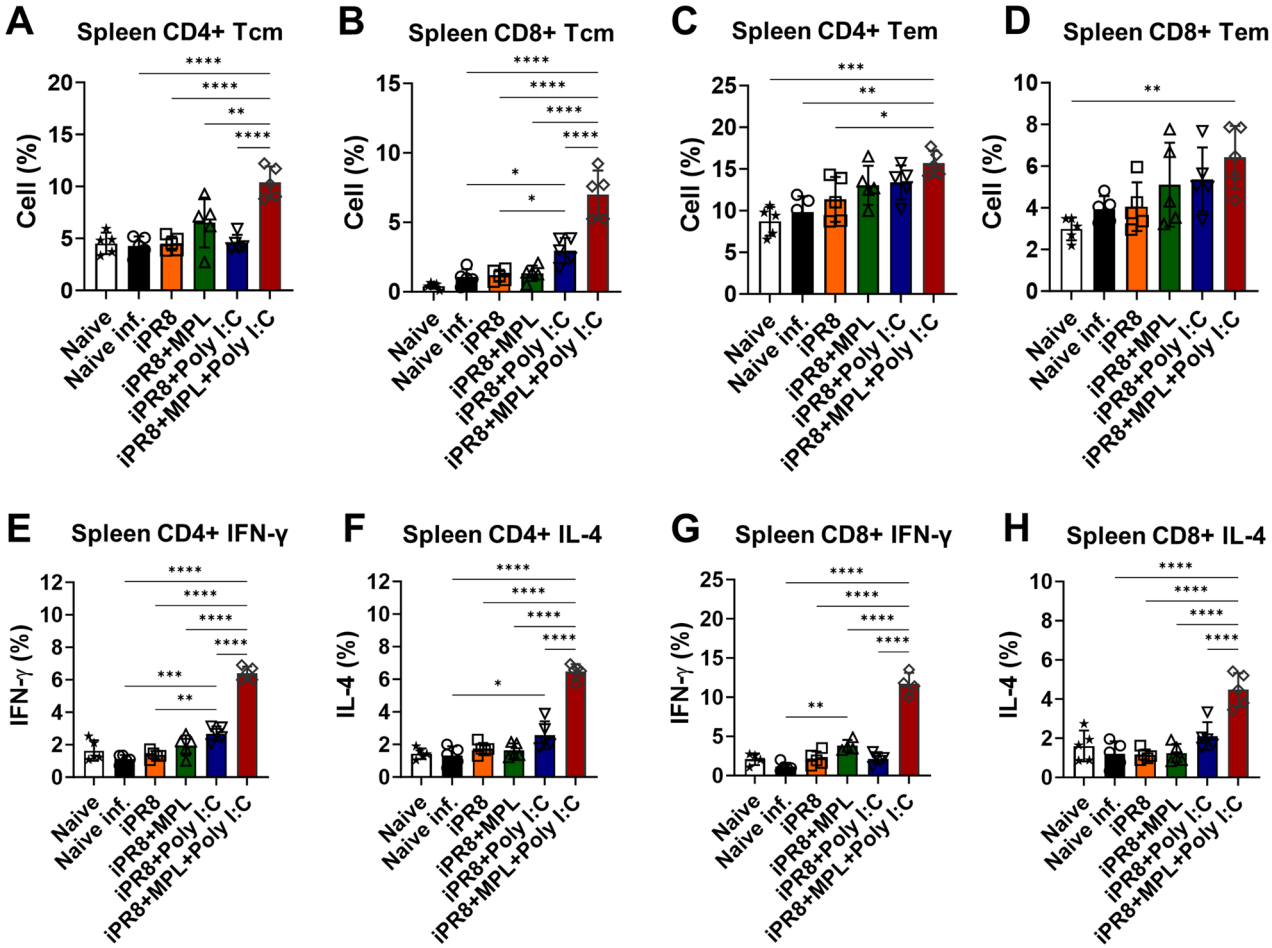
Splenic memory T cell populations and iPR8-specific IFN-γ and IL-4-producing T cells in the immunized mice. Vaccinated mice were infected with a lethal dose of A/PR8 (1.5 × LD_50_) after 3 weeks of vaccination. Splenocytes were harvested 7 days after viral challenge and then stimulated with inactivated PR8 antigen for 5 h. All results are expressed as mean ± standard deviation (SD) with individual dots. Statistical analysis was performed using one-way ANOVA and Tukey’s post-multiple comparison tests. *P < 0.0332, **P < 0.0021, ***P < 0.0002, ****P < 0.0001.

Memory T cells provide protective functions against the invading influenza virus, and high frequencies of antigen-specific memory T cells contribute to the less severe lung diseases^19,20^. Furthermore, after resolving viral invasion, the majority of effector T cells die, leaving a small proportion of long-lived memory T cells contributing to the prevention of reinfection^21^. To test the hypothesis that adjuvants can influence the populations of memory T cells, we examined memory T cells and antigen-specific T cell responses at 7 dpi. The adjuvanted vaccine groups after virus exposure increased the populations of effector memory CD4/CD8 T cells (T_EM_) in the lungs and spleens (Figs. 5C,D and 6C,D), but MPL + poly I:C-adjuvanted vaccination strongly increased the populations of central memory CD4/CD8 T cells (T_CM_) (Figs. 5A,B and 6A,B). Subsequently, we examined the levels of IFN-γ/IL-4-producing T cell populations in the lungs and spleens of the immunized or naïve mice at 7 dpi after homologous A/PR8 antigen stimulation in vitro. Similarly, the cytokine production of T cells was higher in the mice from the MPL + poly I:C-adjuvanted group after antigen stimulation than in the mice from the other groups (Figs. 5E–H and 6E–H).

**Figure 7:**
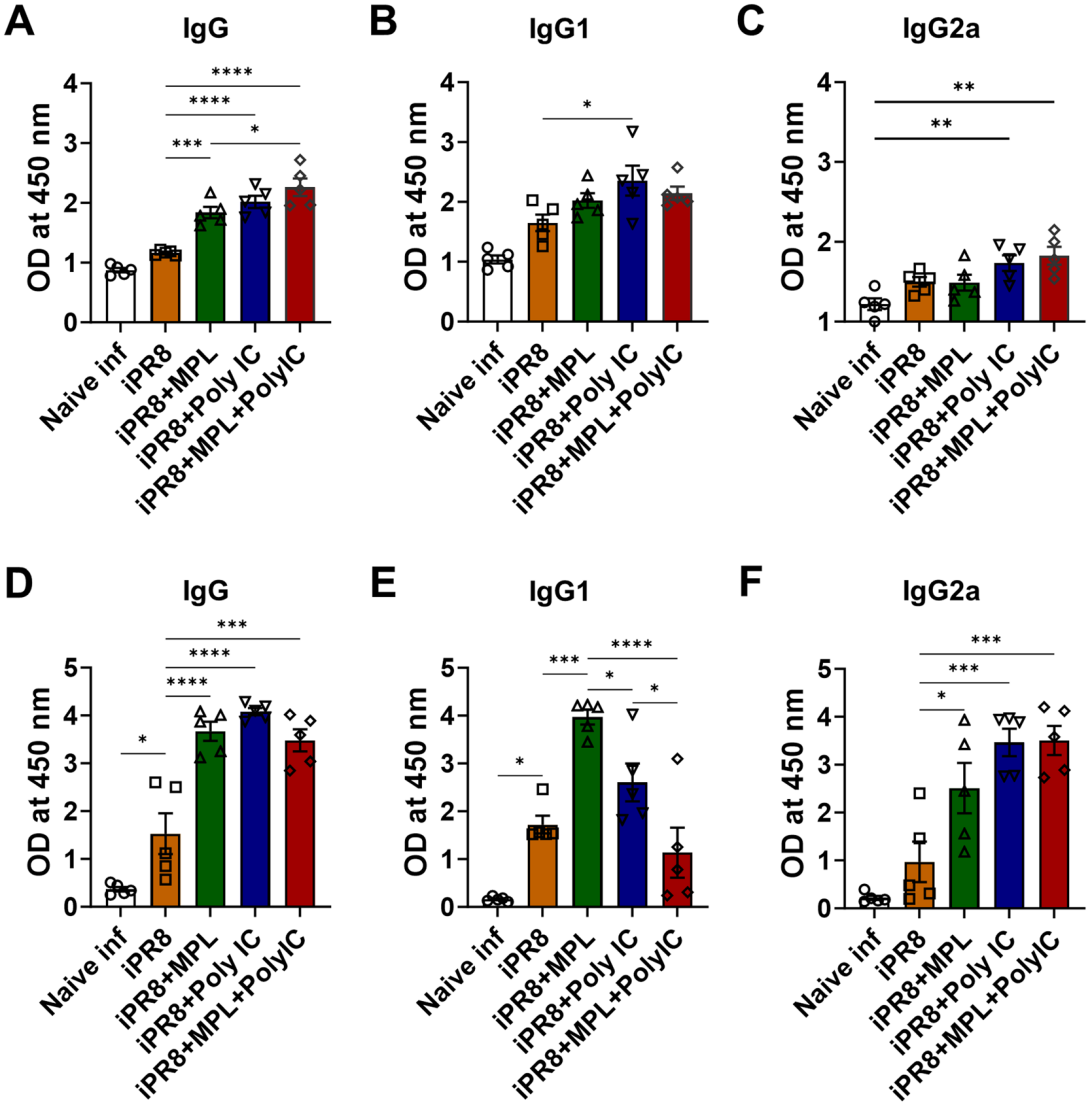
Antigen-specific antibody production of bone marrow and spleen cells from the immunized mice. Bone marrow (**A**–**C**) and spleen cells (**D**–**F**) were harvested at day 7 post virus infection and cultured in iPR8-coated plates for 1 day or 7 days, respectively. IgG, IgG1, and IgG2a ELISA was performed to measure the antigen-specific antibody production. All results are expressed as mean ± standard deviation (SD) with individual dots. Statistical analysis was performed using one-way ANOVA and Tukey’s post-multiple comparison tests. *P < 0.0332, **P < 0.0021, ***P < 0.0002, ****P < 0.0001.

To investigate antibody-producing plasma and memory B cell responses induced by the adjuvanted vaccination, bone marrow and spleen cells were harvested and cultured in antigen-coated plates. MPL + poly I:C-adjuvanted group exhibited significantly enhanced IgG production with higher IgG2a/IgG1 ratio (Fig. 7).

Collectively, these results suggest that the MPL + poly I:C-adjuvanted vaccine was effective in eliciting memory T-cell differentiation and inducing antigen-specific functional T cell populations in addition to memory B cell responses, which provided an efficient protective response against viral invasion.

## Discussion

Apart from COVID-19, influenza infections are among the most urgent infectious diseases to date and responsible for thousands of deaths annually worldwide. Vaccination is a common strategy for preventing the influenza pandemic. Inactivated pathogen vaccines are safer than live attenuated vaccines. Nevertheless, inactivated whole-virus vaccines sometimes lose immunogenicity and do not efficiently induce cellular adaptive immunity^22^. Adjuvants have been introduced to effectively stimulate vaccine antigen-specific immune responses via different pathogen-associated molecular pattern signaling pathways. TLR agonists are widely used as adjuvants that stimulate immune responses by regulating TRIF or MyD88-dependent signals^23–25^. MPL is an attenuated type of lipopolysaccharide that stimulates immune responses by upregulating TRIF/MyD88-dependent signaling pathways, which are licensed for hepatitis B virus vaccine (Fendrix) and human papillomavirus vaccine (Cervarix)^7,26^. Poly I:C is a synthetic double-stranded RNA that effectively triggers immune responses to the influenza virus by promoting the TRIF-dependent signaling pathway, which has been tested in humans^8,26^. The combination of MPL and poly I:C can induce strong innate immune responses and further stimulate antigen-specific antibody production and antigen-specific T cell responses to ovalbumin at high levels^13,14^. In the present study, the inactivated influenza virus (inactivated A/PR8 (H1N1)) vaccine combined with either MPL, poly I:C, or MPL + poly I:C at low doses exerted additive effects on enhancing virus-specific IgG, IgG1, and IgG2a isotype antibody production; preventing weight loss disease, pro-inflammatory cytokine release and inflammatory immune cell infiltration into the infection site upon viral infection. Additionally, the MPL + poly I:C-adjuvanted iPR8 vaccine elicited robust memory immunity, as demonstrated by the increased levels of antigen-specific antibody production by plasma and memory B cells, frequencies of central/effector memory T cells and antigen-specific cytokine producing memory T cell responses in the secondary lymphoid organs. These results suggest that the inactivated vaccine adjuvanted with the combination of TLR3 and 4 agonists (MPL and poly I:C, respectively) showed effective protection against viral infection with better antigen-specific memory responses.

In the present study, the adjuvanted groups showed strong humoral immune responses with high levels of virus-specific antibodies (Fig. 1A,B). This result might be related to the pre-existence of memory CD4/CD8 T cell populations in the lungs and spleens upon viral infection^21,27^. In addition to serum antibody responses, neutralizing antibody (NAb) binding to HA plays a vital role in preventing the interaction between virus sialic acid receptors and the host cell^27–31^. The MPL + poly I:C- or individual poly I:C-adjuvanted iPR8 was effective in inducing high levels of HAI titers against A/PR8 (H1N1) (Fig. 1C), which contributed to viral clearance upon infection, as demonstrated by the very low viral titers (Fig. 2C). Moreover, NAbs not only function against the same strain of influenza but also accelerate the clearance of different serotypes of influenza^32^. Thus, we determined the A/HK (H3N2)-specific IgG and HAI titers in the sera and IgG production by plasma cells derived from bone marrow cells after immunization and homologous virus challenge (Supplementary Fig. 3). The MPL + poly I:C-adjuvanted vaccine significantly enhanced heterosubtypic A/HK (H3N2)-specific immune responses such as binding IgG, HAI activities, and antibody-secreting plasma cells, suggesting that the combination adjuvant may elicit cross-protection against different influenza strains.

The inflammatory cytokines and chemokines released into infection sites play vital roles in the recruitment of immune cells against invading pathogens^33^. However, excessive recruitment of cell infiltrates and high levels of pro-inflammatory cytokines contribute to immune-mediated damage, which exacerbates illness outcomes due to influenza infection^34,35^. AMs provide a crucial defense against pathogens during infection; lung-resident AMs contribute to the protection of alveoli from inflammation following viral infection^36^. In the present study, high levels of lung-resident AMs and moderately low frequencies of inflammatory infiltrates into the lungs were observed in the mice from the MPL + poly I:C-adjuvanted group after influenza infection (Fig. 4), and these mice had no BW losses and 100% survival rates (Fig. 2A,B).

An efficient response of the Th1 and Th2 subsets contributes to the control of viral infections. However, high levels of IL-4, correlated with the induction of the Th2 response, may delay viral clearance^37^. Eosinophils, which are recruited in response to IL-4 and Th2 expression, have been associated with local allergic responses that can damage lung histopathology^21,37^. Though virus infection usually induces Th1 immune responses due to their intracellular activity, inactivated influenza virus vaccine was previously reported to induce Th2-biased immune responses^38,39^. As a result, immunization of whole-virus inactivated vaccine may present serious adverse events and lower vaccine efficacy. In the present study, the inactivated vaccine adjuvanted with the combination of MPL and poly I:C provided enhanced protection and reduced inflammation at the infection site (Fig. 2), but it also possibly increased Th1-polarized immune responses, as evidenced by the increased levels of antigen-specific IFN-γ and IL-4 (Fig. 5E–H), which provided sufficient protection against illness severity due to viral infection^40^.

Lung-resident memory CD8 T cells are primarily localized in the epithelial layers and can mediate rapid responses to invading pathogens. Additionally, lung-resident memory T cells mainly comprise tissue-resident memory T cells (T_RM_ cells), which play a predominant role in protective immunity^41^. Among functional CD8 T cell subsets, effector CD8 T cells protect against influenza by blocking viral replication. High levels of CD8^+^IFN-γ^+^ were reported to be correlated with an elimination of symptoms, reduction of illness severity, and clearance of virus in infected patients^19,42–44^. Aside from CD8 T cells, memory CD4 T cells contribute directly to viral clearance by interacting with antigen-presenting cells, helping virus-specific antibody-producing B cells, and improving CD8 T cell functionality^21^. CD4 T cell-derived IFN-γ directly downregulates T-bet expression on CD8 T cells, leading to T_RM_ formation during influenza infection^45^. Moreover, IL-4-producing T cells induce the differentiation of CD44^high^CD62L^high^ central memory cell-like CD8 T cells after viral infection, which is a robust effector cytokine that controls chronic viral infection; therefore, virus-specific IL-4-induced innate CD8 T cells are potentially used in T-cell therapy^46^. In the present study, the MPL + poly I:C-adjuvanted iPR8 vaccine induced higher levels of CD8/CD4 T_CM_ and T_EM_ residing in the lungs compared with the individual adjuvanted and nonadjuvanted vaccines (Fig. 5), which conferred complete protection from severe disease outcomes upon viral infection. Furthermore, the vaccine with the combination adjuvant elicited more functional T-cell immunity, such as antigen-specific IFN-γ and IL-4 production, than the other vaccines. In addition to the homologous protection, local CD4 and CD8 T cells confer cross-protection against different serotypes of influenza viruses^47^, supporting the cross-protective efficacy of the MPL + poly I:C-adjuvanted vaccine.

T cells residing in secondary lymphoid tissues are important for providing long-lasting antiviral responses. Most effector CD4/CD8 T cells die after viral infection, but only a small number of memory T cells persist in the long term at the infection site^48–50^. Therefore, the memory CD4/CD8 T cells located in secondary lymphoid organs appear to play a critical role in the early clearance of the virus upon reinfection. In addition, central memory T cells have a unique functional attribute of migrating to the infection sites following differentiation into effector T cells producing antiviral cytokines, resulting in rapid and effective antiviral responses due to virus reinfection^51^. Our data revealed that the MPL + poly I:C-adjuvanted vaccine was not only effective in enhancing splenic T_CM_ and T_EM_ cell populations but also in efficiently inducing T cells to release significantly high levels of antigen-specific effector cytokines (IL-4 and IFN-γ).

In summary, the results of this study showed that the iPR8 vaccine adjuvanted with the combination of MPL and poly I:C enhanced antigen-specific antibody production, memory T cell responses, and protection against homologous influenza infection. In addition, the MPL + poly I:C-adjuvanted vaccine showed potential cross-reactive IgG and HAI activities. Thus, this study suggests the combination of MPL and poly I:C as an adjuvant for influenza vaccines. However, further studies are warranted to elucidate the mechanisms by which the MPL + poly I:C adjuvant induces cross-protection.

## Methods

### Mice and reagents

Female BALB/c mice (n = 5 each group, OrientBio Co., Gyeonggi, Korea) were used in this study. The mice were 8–10 weeks old at the time of priming immunization. The mice were fed standard chow diet, provided with water ad libitum, and maintained under a 12 h light/dark cycle at 22 ± 2 °C. The mice were anesthetized with isoflurane and O_2_ gas mixture for the experimental procedures. All animal experiments confirmed to the International Guide for the Care and Use of Laboratory Animals and were approved by the Institutional Animal Care and Use Committee (IACUC) of Jeju National University, South Korea (protocol number: 2021–0051). All animal experiments were conducted in accordance with relevant regulations and guidelines, and reported in accordance with ARRIVE guidelines.

MPL and poly I:C were purchased from InvivoGen (San Diego, CA). All reagents were prepared in accordance with the manufacturer’s instructions. A/PR8 and A/HK grown in the allantoic cavity of embryonated chicken eggs were used to prepare an inactivated virus vaccine with 1% neutral buffered formalin overnight at 4 °C and then concentrated by ultracentrifugation (30,000 rpm, 1 h). The inactivated virus pellets were resuspended in phosphate-based saline (PBS) and then stored at − 80 °C until use. Live A/PR8 was used as the homologous challenge.

### Immunization and viral infection

BALB/c mice (n = 10/group) were immunized intramuscularly with inactivated PR8 (iPR8) vaccine (1 μg) alone or in combination with MPL (1 μg), poly I:C (10 μg), or MPL + poly I:C (1 μg + 10 μg, respectively). Vaccinations were administered two times (prime and boost) with a 3 weeks interval, and sera were collected 2 weeks after each immunization. At 6 weeks after the last immunization, the naïve and vaccinated mice were challenged intranasally with a lethal dose (1.5 × LD50) of A/PR8. Changes in body weight (BW) and survival rates were monitored for 14 days after the challenge. In accordance with IACUC guidelines, mice displaying more than 20% BW loss were considered to have reached the endpoint and humanely euthanized to minimize pain.

### Enzyme-linked immunosorbent assay for antibody and cytokine productions

Immune sera were collected 2 weeks after each vaccination, and antigen-specific antibody levels were determined using enzyme-linked immunosorbent assay (ELISA). ELISA plates were coated with either inactivated A/PR8 (600 ng/well) or A/HK (H3N2) overnight at 4 °C and blocked with PBS containing 3% bovine serum albumin and 0.05% tween20. The plates were added with serially diluted immune sera, incubated, and then washed. Horseradish peroxidase-labeled secondary antibodies were used to detect antigen-specific IgG, IgG1, and IgG2a antibodies. Tetramethylbenzidine was used as a substrate, and absorbance was obtained at an optical density of (OD) 450 nm by using an ELISA reader.

The levels of cytokines and chemokines in lung extracts obtained from immunized mice were measured using interleukin (IL)-1β, IL-6, and tumor necrosis factor (TNF)-α kits from Invitrogen (Waltham, MA, USA) and interferon IFN-γ, monocyte chemoattractant protein-1 (CCL2/MCP-1), and C–C motif chemokine ligand 5 (CCL5) kits from R&D Systems (Minneapolis, MN, USA) in accordance with the manufacturers’ instructions.

### Lung viral titration

Lungs of the A/PR8-infected BALB/c mice were harvested at 7 days postinfection (dpi) and minced mechanically with 2 mL of Roswell Park Memorial Institute (RPMI) 1640 medium (Fisher Scientific, Corning, NY, USA) per lung. Lung extracts were collected by centrifugation, and 200 μl of extracts was saved for measurement of lung viral titers. Embryonated chicken eggs were prepared for 9–10 days to be inoculated with diluted lung extracts with PBS, and viral titers were determined by HA assay of the allantoic fluids collected after 3 days of incubation. Viral titers at 50% egg infection dose (EID_50_)/ml were evaluated using the Reed and Muench method^52^.

### HA inhibition (HAI) assay

The ability of immune sera to inhibit HA was estimated by performing an HAI assay after boost and challenge infection. Immune sera collected from immunized BALB/c mice were inactivated by incubation at 56 °C for 30 min and then treated with the receptor-destroying enzyme (RDE, Sigma-Aldrich) for 18 h at 37 °C. Subsequently, sera were serially diluted in PBS and incubated with 4 HA units of A/PR8 (H1N1) or 4 HA units of A/HK (H3N2) for 30 min at room temperature (RT), and then 0.5% chicken red blood cells were added to determine HAI titers.

### Memory B cell response

The A/PR8 or A/HK (H3N2)-coated plates were blocked with 10% fetal bovine serum containing RPMI 1640 medium for 1 h at RT to measure the memory B cell response to influenza virus. Bone marrow and spleen cells derived from the infected mice at 7 dpi were seeded at a density of 2 × 10^6^ cells/ml onto the plates and then incubated at 37 °C for 1 day or 5 days, respectively. Anti-mouse IgG, IgG1, IgG2a antibodies were used to detect influenza-specific antibodies secreted by antibody producing B cells.

### Flow cytometry

The inflammatory cell infiltration in the respiratory tract was investigated by collecting lung samples at 7 dpi and staining single cells with antibodies specific for CD45 (clone 30-F11), CD3 (clone 17A2), CD11b (clone M1/70), CD11c (clone N418), F4/80 (clone BM8), Ly6c (clone AL-21), major histocompatibility complex (MHC) class II (clone I-A/I-E), SiglecF (clone E50-2440), B220 (RA3-6B2), and live/dead-amcyan (LIVE/DEAD™ Fixable Aqua Dead Cell Stain Kit) after blocking the Fc receptor.

Lungs and spleens were harvested and stained with antibodies specific for CD45, CD3, CD4 (clone RM4.5), CD8a (clone 53-6.7), CD44 (clone IM7), CD62L (clone MEL-14), and live/dead cells to explore the memory T cells in lung and spleen cells.

Intracellular cytokines (IL-4 [clone 11B11] and IFN-γ [clone XMG1.2]) of CD4 and CD8 T cells residing in the lung and spleen cells were stained after 5 h stimulation with inactivated A/PR8 to estimate the A/PR8 specific-IL-4/IFN-γ production by CD4 and CD8 T cells. Golgi stop (monensin, a protein transport inhibitor) was added to each sample during the stimulation. Intracellular cytokine staining was performed using the fixation/permeabilization solution kit (BD Biosciences) in accordance with the manufacturer’s instructions.

The phenotypes of the acquired cells were analyzed and gated by marker expressions on cells: alveolar macrophages (AMs): CD45^+^CD11b^−^CD11c^+^F4/80^+^; neutrophils: CD45^+^CD11b^+^Ly6c^low^F4/80^−^; eosinophils: CD45^+^CD11b^+^SiglecF^+^; total dendritic cells (DCs): CD45^+^F4/80^−^CD11c^+^MHCII^high^; plasmacytoid DCs (pDCs): CD45^+^F4/80^−^CD11c^+^MHCII^high^B220^+^; CD11b^+^DCs: CD45^+^F4/80^−^CD11c^+^MHCII^high^CD11b^+^; CD4 naïve T cell: CD45^+^CD3^+^CD4^+^CD44^−^CD62L^+^; CD8 naïve T cell: CD45^+^CD3^+^CD8^+^CD44^−^CD62L^+^; CD4 central memory T cell (T_CM_): CD45^+^CD3^+^CD4^+^CD44^+^CD62L^+^; CD8 T_CM_: CD45^+^CD3^+^CD8^+^CD44^+^CD62L^+^; CD4 effector memory T cell (T_EM_): CD45^+^CD3^+^CD4^+^CD44^+^CD62Lˉ; CD8 T_EM_: CD45^+^CD3^+^CD8^+^CD44^+^CD62Lˉ. The gating strategies of inflammatory cells and T cells are shown in Supplementary Figs. 4 and 5.

### Statistical analysis

All results are presented as the mean ± standard error of the mean (SEM). Statistical significance was determined using 1-way ANOVA or 2-way ANOVA and considered at *P* < 0.05. All data were analyzed using GraphPad Prism statistical software Inc. (San Diego, CA, USA).

## Supplementary Information

Supplementary Information.

## Data Availability

All relevant data generated during this study are available upon reasonable request made to correspondence authors.
