# Supplementary material for: Adjuvant effects of combination monophosphoryl lipid A and poly I:C on antigen-specific immune responses and protective efficacy of influenza vaccines
Source: Sci Rep. 2023 Jul 28;13:12231. doi: 10.1038/s41598-023-39210-6 (PMC10382554; doi:10.1038/s41598-023-39210-6)
Supplement: Supplementary file 1 — Supplementary Information. [file 41598_2023_39210_MOESM1_ESM.pdf]

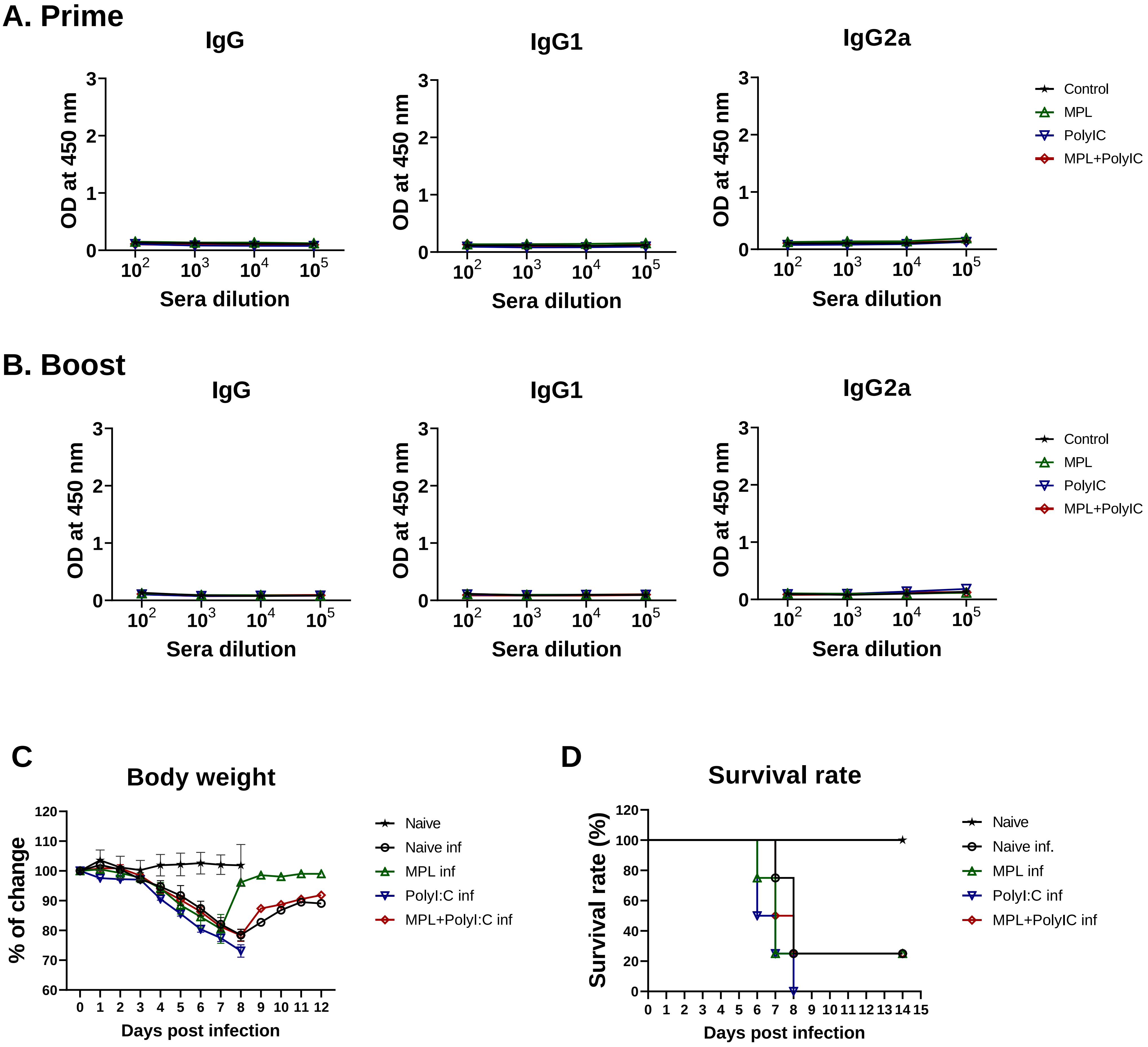

Supplementary figure 1. Adjuvant itself could not provide protection against the virus infection. Balb/c mice were immunized with MPL, Poly I:C, or MPL+Poly I:C without vaccine antigen to evaluate the protective effects of adjuvant. Immune sera were taken 2-weeks after prime and boost immunization to measure iPR8-specific antibody levels. Three-weeks post the last immunization, the mice were infected with 1.5×LD50 of A/PR8 and monitored body weight changes and survival rates for 14 days. All results were shown in mean ± standard deviation (SD).

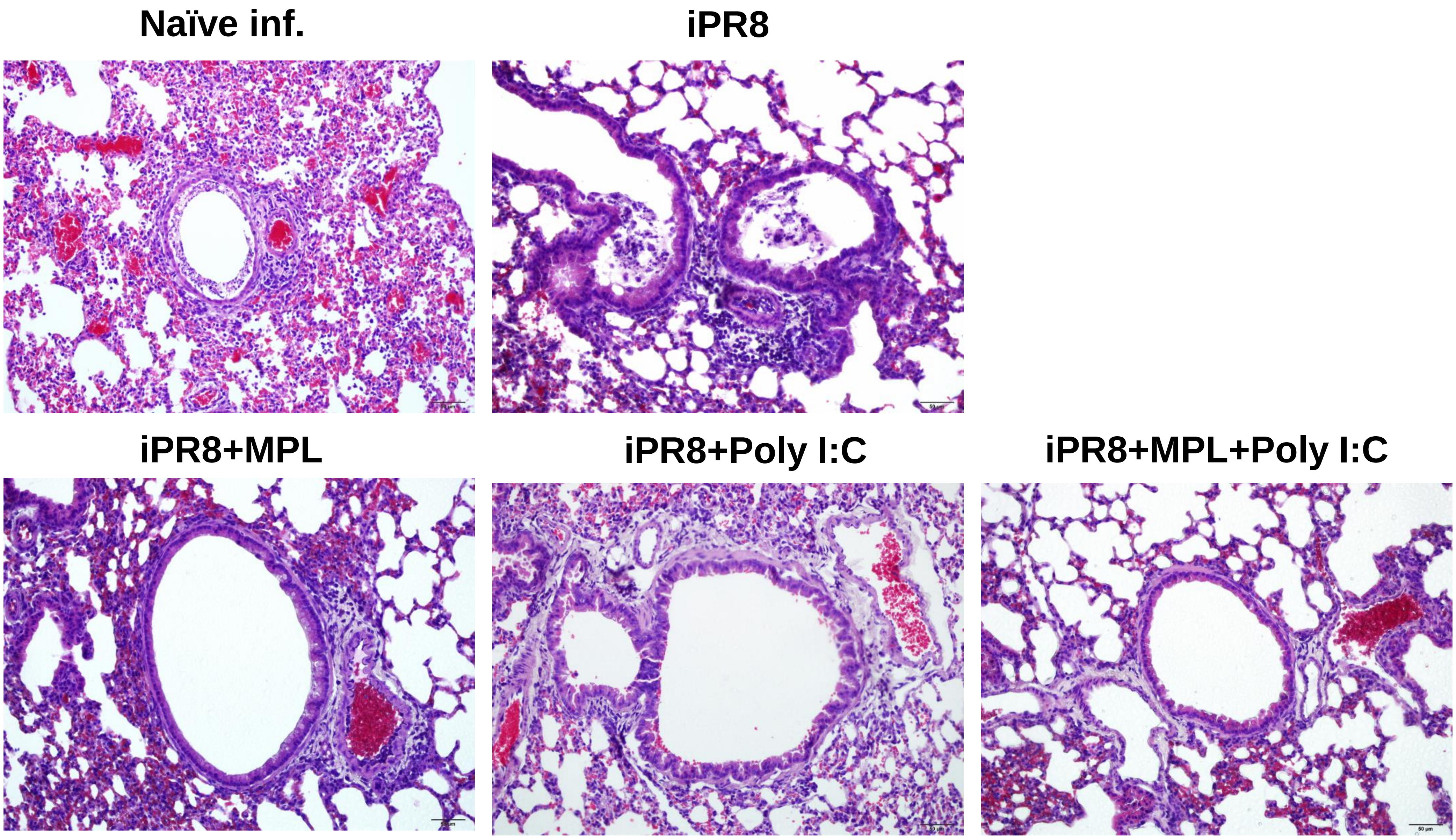

Supplementary figure 2. Lung histopathology. Intact lungs were collected at day 7 post infection, fixed, processaed, and stained with hematoxylin and eosin (H&E). Scale bars indicate 50 µm.

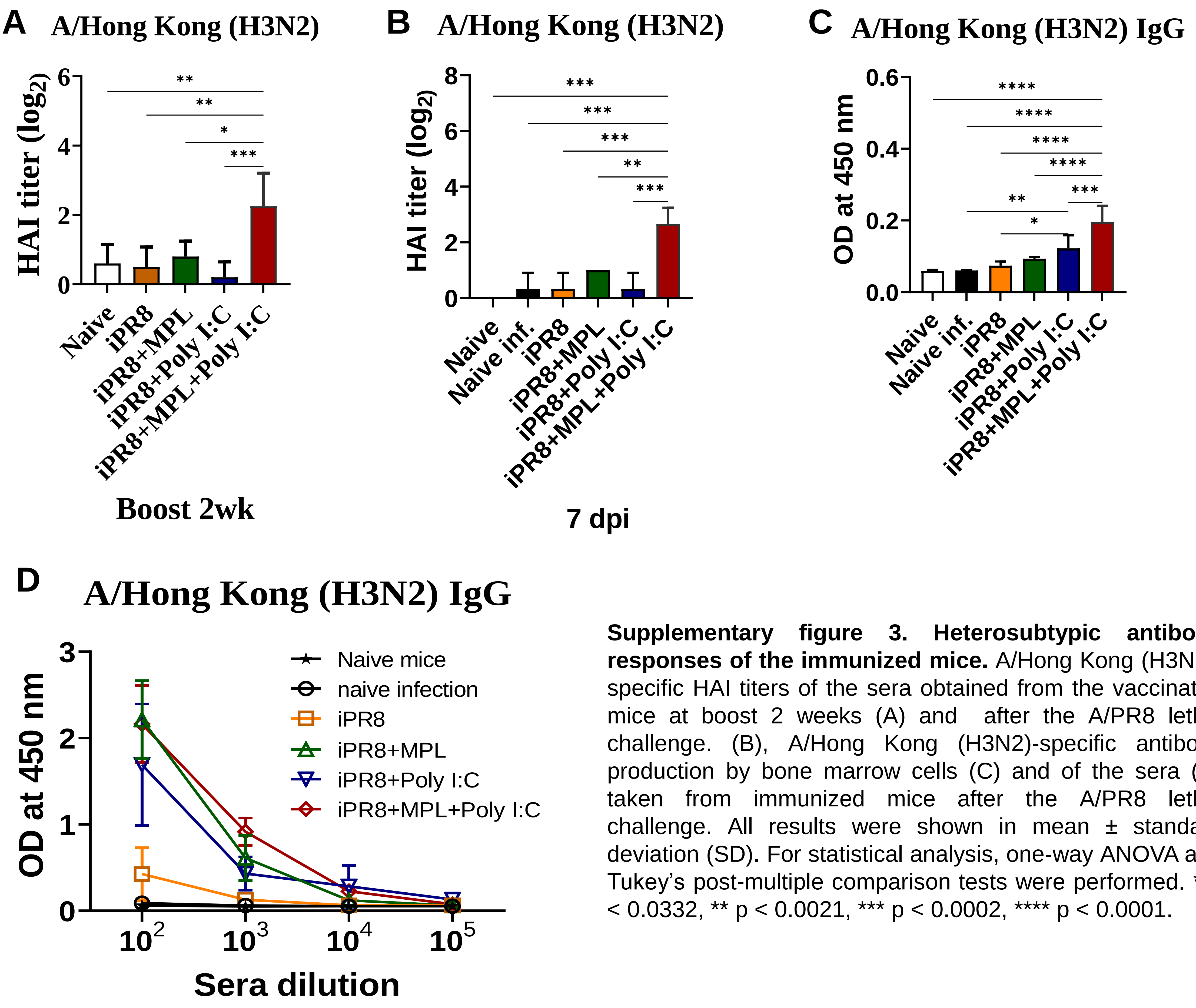

Supplementary figure 3. Heterosubtypic antibody responses of the immunized mice. A/Hong Kong (H3N2)-specific HAI titers of the sera obtained from the vaccinated mice at boost 2 weeks (A) and after the A/PR8 lethal challenge. (B), A/Hong Kong (H3N2)-specific antibody production by bone marrow cells (C) and of the sera (D) taken from immunized mice after the A/PR8 lethal challenge. All results were shown in mean ± standard deviation (SD). For statistical analysis, one-way ANOVA and Tukey's post-multiple comparison tests were performed. * $p < 0.0332$, ** $p < 0.0021$, *** $p < 0.0002$, **** $p < 0.0001$.

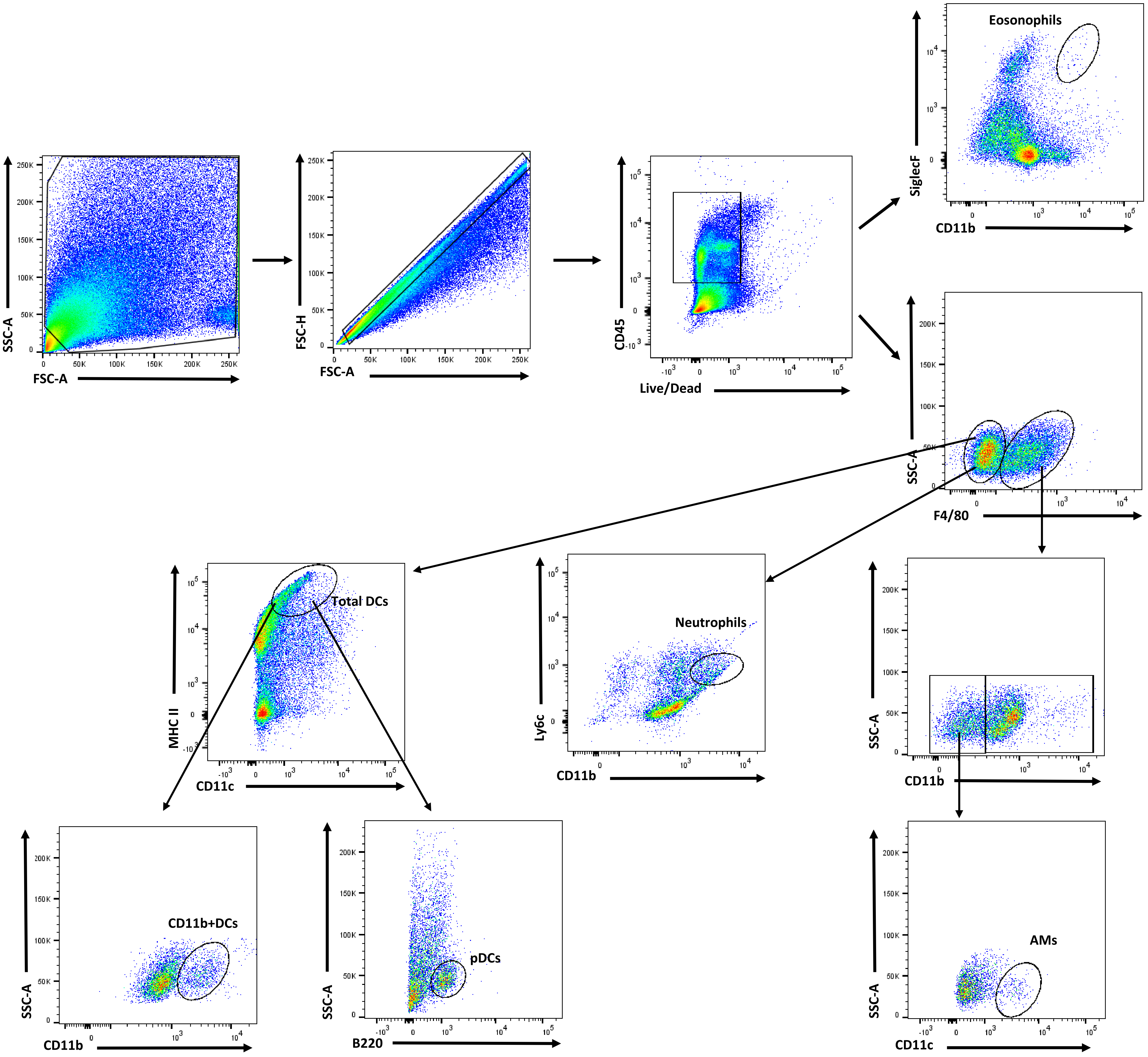

Supplementary figure 4. Flowcytometry gating strategy. eosonophils: $CD45^+CD11b^+SiglecF^+$; aveolar macrophages (AMs): $CD45^+F4/80^+CD11b^-CD11c^+$; macrophages: $CD45^+F4/80^+CD11b^+$; neutrophils: $CD45^+F4/80^-CD11b^+Ly6c^{low}$; total DCs: $CD45^+F4/80^-CD11c^+MHCII^{high}$; plasmacytoid DCs (pDCs): $CD45^+F4/80^-CD11c^+MHCII^{high}B220^+$; CD11b+DCs: $CD45^+F4/80^-CD11c^+MHCII^{high}CD11b^+$;

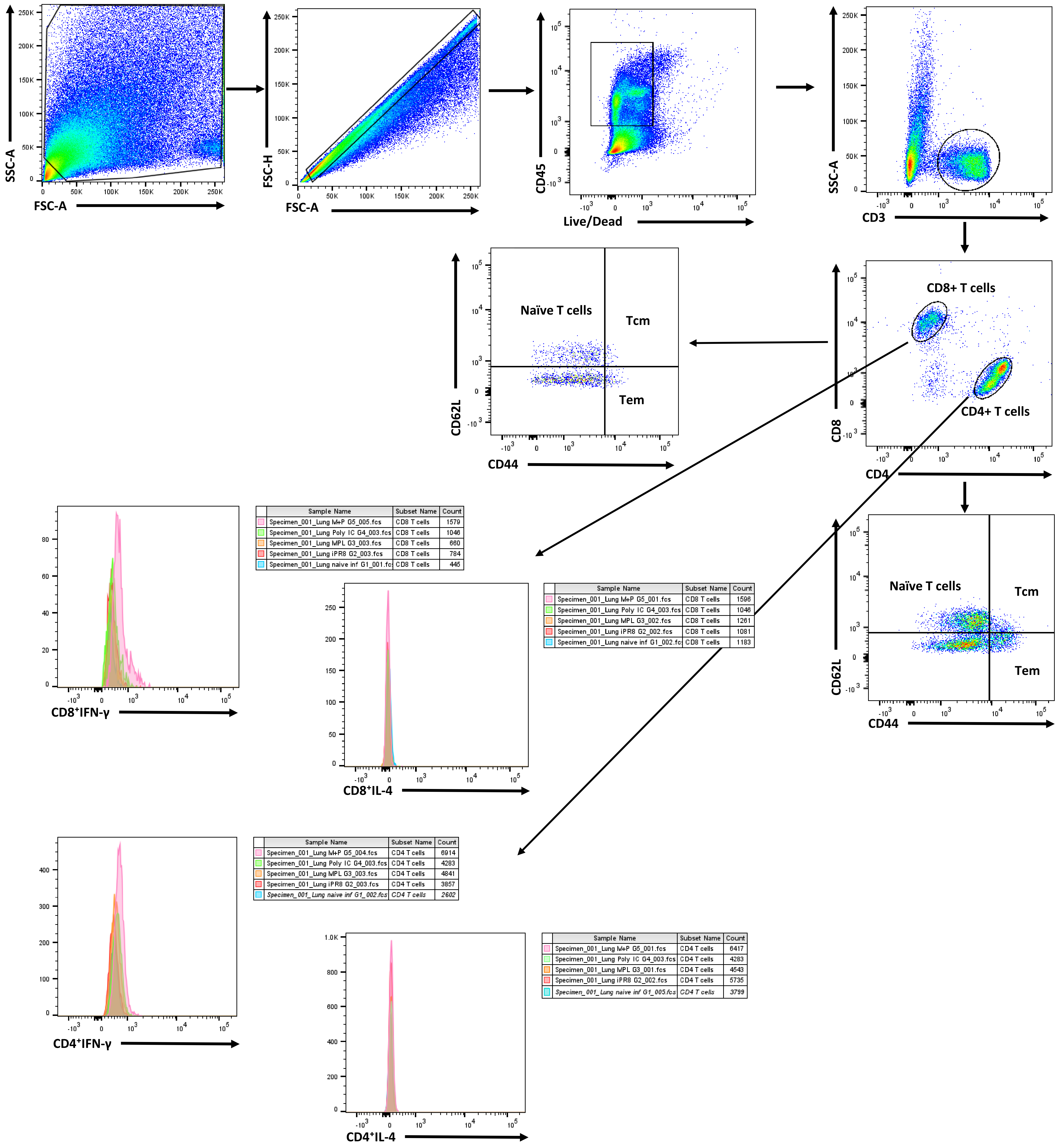

Supplementary figure 5. The phenotypes of the T cells and cytokine-producing T cells. CD4 naïve T cell: $CD45^+CD3^+CD4^+CD44^-CD62L^+$; CD8 naïve T cell: $CD45^+CD3^+CD8^+CD44^-CD62L^+$; CD4 central memory T cell (T_{CM}): $CD45^+CD3^+CD4^+CD44^+CD62L^+$; CD8 T_{CM}: $CD45^+CD3^+CD8^+CD44^+CD62L^+$; CD4 effector memory T cell (T_{EM}): $CD45^+CD3^+CD4^+CD44^+CD62L^-$; CD8 T_{EM} : $CD45^+CD3^+CD8^+CD44^+CD62L^-$.IFN-$\gamma^+CD8^+$ T cell: $CD45^+CD3^+CD8^+$IFN-γ^+; IL-4^+CD8^+ T cell: $CD45^+CD3^+CD8^+$IL-4^+ ; IFN-γ^+CD4^+ T cell: $CD45^+CD3^+CD4^+$IFN-γ^+; IL-4^+CD4^+ T cell: $CD45^+CD3^+CD8^+$IL-4^+
